# Supplementary material for: Transcriptomics informed discovery of developmentally essential transcription factors
Source: Biol Open. 2025 Dec 2;14(11):bio062354. doi: 10.1242/bio.062354 (PMC12714135; doi:10.1242/bio.062354)
Supplement: Supplementary information [file biolopen-14-062354-s1.pdf]

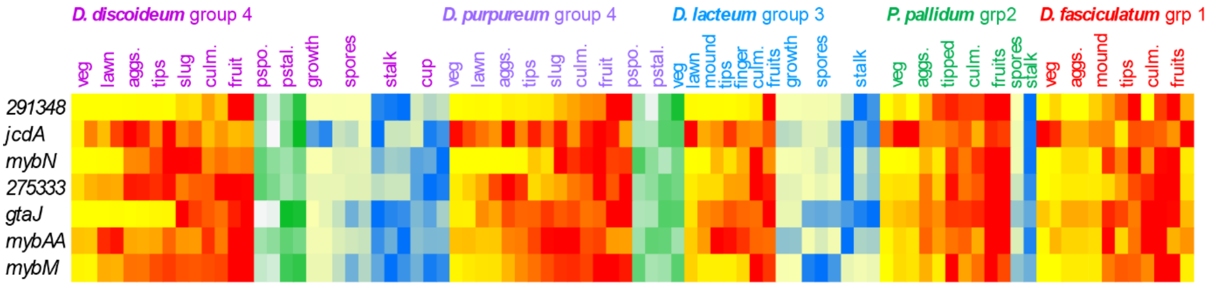

Fig. S1. Transcription profiles of target genes

Developmental and cell-type specific expression profiles of the targeted TF genes and their orthologs across the 4 dictyostelid taxon groups. Data were retrieved from published RNAseq experiments (Forbes et al., 2019; Gloeckner et al., 2016; Kin et al., 2018; Parikh et al., 2010) and shown as heatmaps of relative transcript levels during development (yellow-red: 0–1 fraction of maximum value), in prespore or prestalk cells (white-green: 0–1 fraction of summed reads), or in vegetative, spore, stalk and cup cells (yellow-blue: 0–1 fraction of maximum value, except *Polyspondylium pallidum a.k.a. Heterostelium album*, fraction of summed reads). Multiple blocks for the same developmental stage or cell-type represent replicate biological experiments.

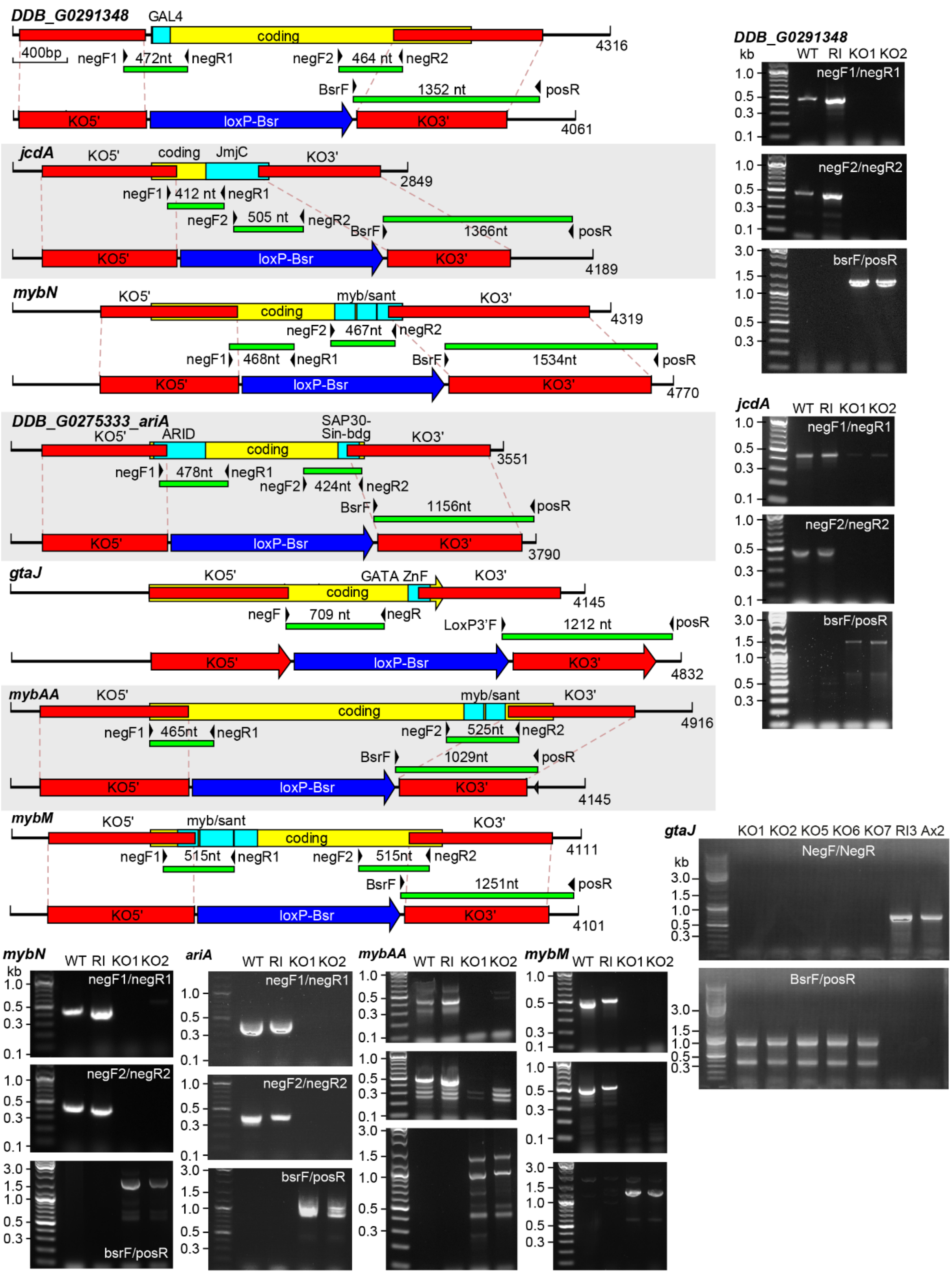

Fig. S2. Knock-out constructs and diagnoses

A. The schematics show genomic fragments of the target TF genes before and after homologous recombination with the knock-out construct, with the positions of the signature DNA binding domain(s) of the genes in light blue, the loxP-Bsr resistance cassette in dark

blue, the primers used for diagnosis as black triangles and the fragments they ampified in green with their sizes.

B. Gel images of the diagnostic PCRs performed on genomic DNAs of wild-type (WT) cells, random vector integrants (RI) clones and knock-out (KO) clones. NegF/NegR primer combinations should only yield bands from WT and RI gDNAs, the BsrF and LoxP3'F primers combined with the gene specific posR primer should only yield bands in KO gDNAs. Primer sequences are listed in Table S1.

Biology Open • Supplementary information

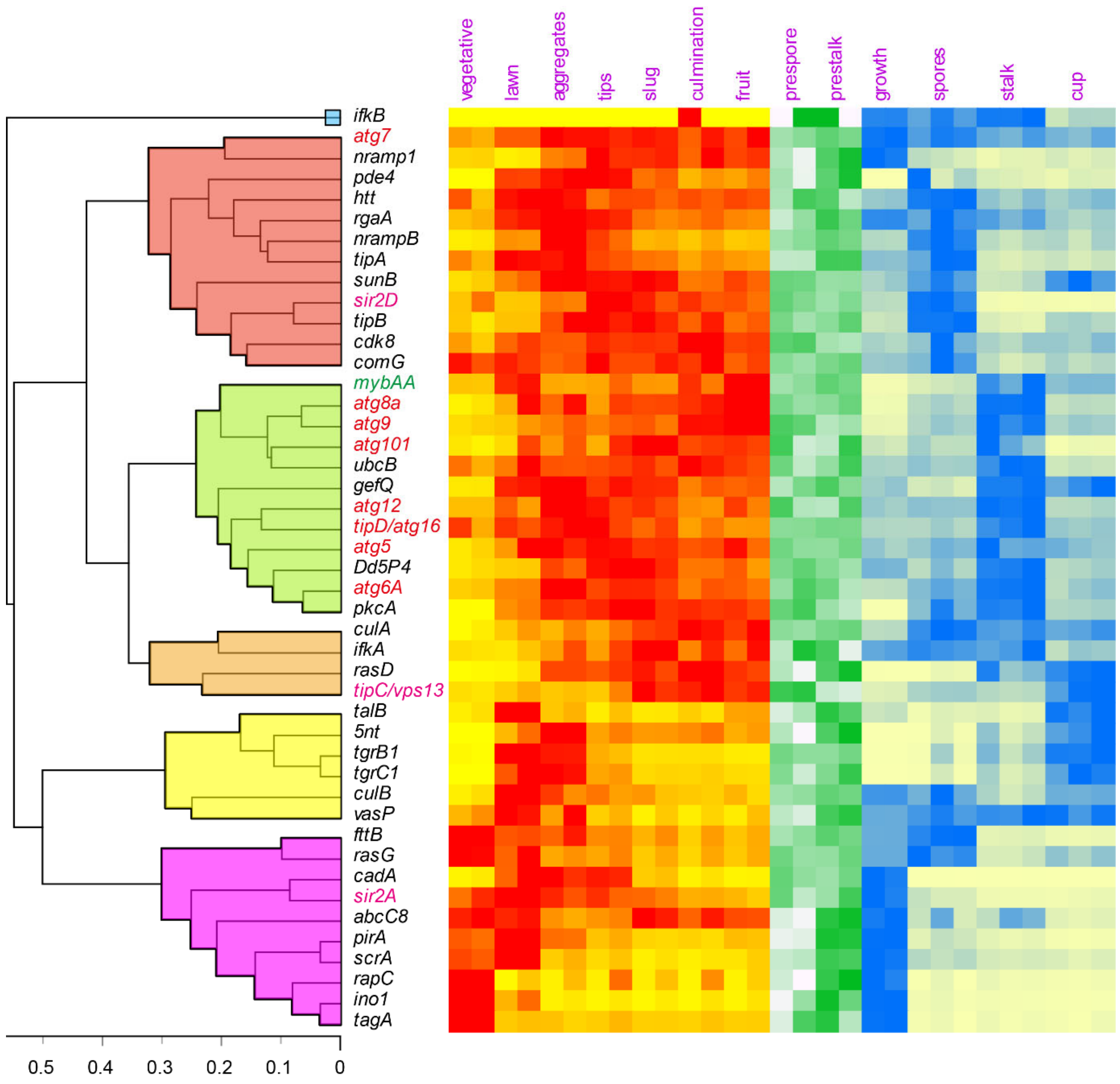

Fig. S3. Expression profiles of genes that suppress multi-tipped phenotypes Knock-out mutants with multi-tipped phenotypes were isolated from the monthly updated mutant phenotype listing in Dictybase http://dictybase.org/Downloads/developmental-mutants.html (Fey et al., 2019). The expression profiles of *mybAA* and the mutant genes were retrieved from published developmental and cell-type specific RNAseq experiments (Kin et al., 2018; Parikh et al., 2010) and subjected to hierarchical clustering using Orange (Demsar et al., 2013). Multiple blocks for the same developmental stage or cell-type represent replicate experiments. Pearson correlation between expression profiles was used as the distance metric and cluster components were ordered by average linkage (Spearman correlation yielded a similar result). The hierarchical tree is shown next to the relative expression levels of the genes. Mutant genes that also cause autophagy defects are shown in red font and *mybAA* in green font.

Biology Open • Supplementary information

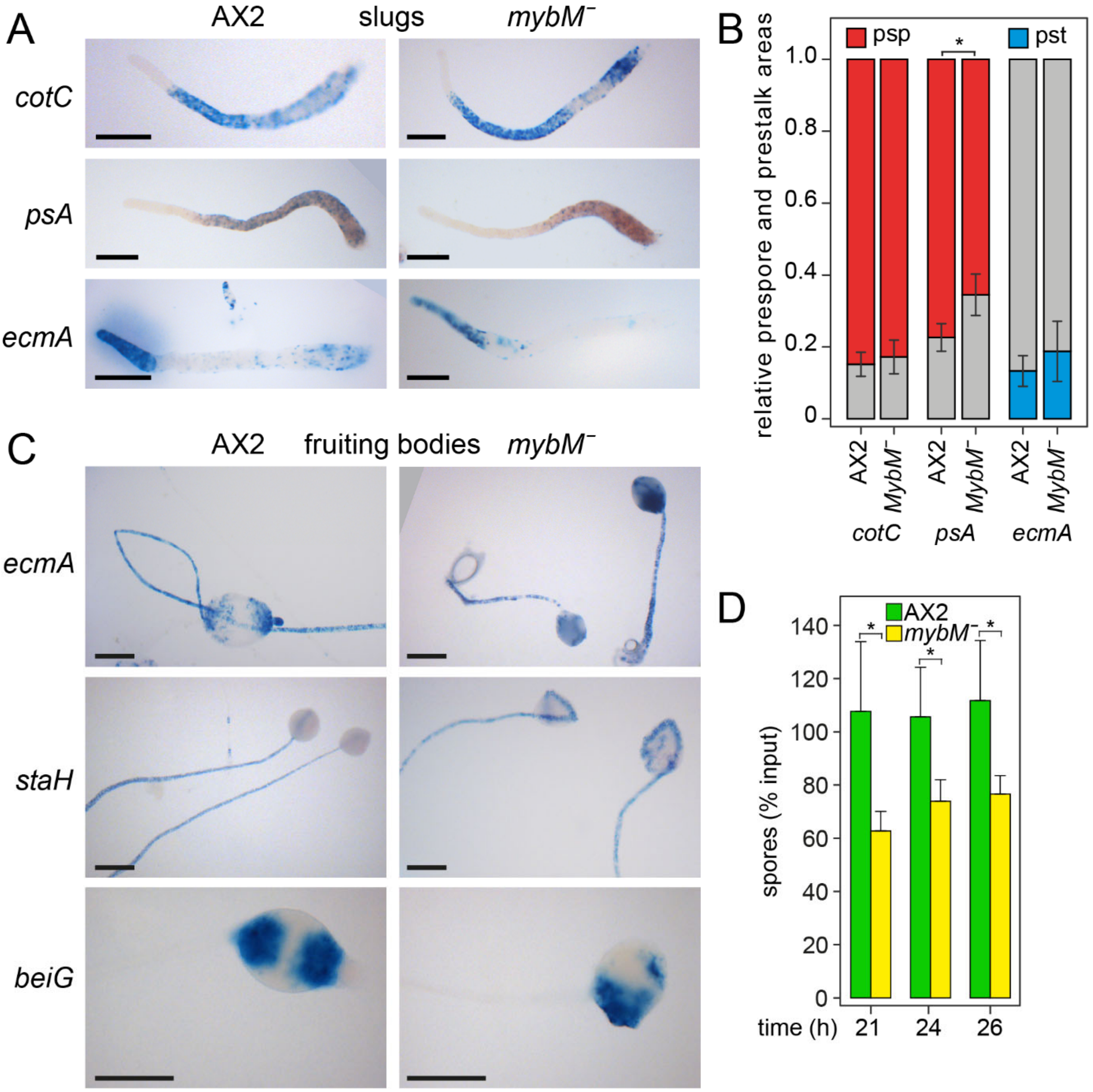

Fig. S4. Cell type markers and sporulation efficiency in *mybM⁻*

A. *Migrating slugs.* AX2 and *mybM⁻* cells were transformed with fusion constructs of *lacZ* with stalk (*ecmA*) and spore (*psA* and *cotC*) promoters. Cells were developed overnight under unilateral light and slugs were fixed and stained with X-gal. Bar:100 µm.

B. *Cell type proportions*. Unstained and X-gal stained areas of migrating slugs were measured from images as in panel A with ImageJ. Data are expressed as the ratio of stained and unstained to total area and represent the mean and SD of 9 slugs. Significant differences between AX2 and *mybM⁻* are indicated by * for P<0. 001.

C. *Fruiting bodies*. AX2 and *mybM⁻* cells transformed with stalk (*ecmA* and *staH*) and cup (*beiG*) promoter fusions with *lacZ* were developed to fruiting bodies, fixed and stained with X-gal. Bar:100 µm.

D. *Sporulation efficiency.* 3x10^6 cells were plated on 1 cm^2 filters and developed into fruiting bodies for the indicated periods, when the spores were washed off and counted. Data are expressed as percentage of counted cells and represent means and standard deviations from 3 experiments with 4 replicate counts. Significant differences between AX2 and *mybM⁻* are indicated by * for P<0. 001.

Table S1. Oligonucleotide primers used in this work

Name	Sequence	site
291348-5'f	AAAGGTACCCACACATAAAAAGTAGATGATTTACG	KpnI
291348-5'r	TTTAAGCTTCGACCTATTTCATGAGTTTAGTG	HindIII
291348-3'f	CACGGATCCCATATTCAATGGAATCAACAAATGG	BamHI
291348-3'r	TAAGCGGCCGCGTGAAGATAGTGATATTGAGGCAA	NotI
291348_negF1	TGGAATGAAGCCAATAATTAATT	
291348_negR1	TGTGATTGTAATTACTTTGACTG	
291348_negF2	TCAAATTGTTCCAAACCTATTAC	
291348_negR2	GTTGAATATGGGTATGGATTTGA	
291348_posR	CAAGTTTTTCACAATCATTCACCACCTTTGGCAC	
jcdA-5'f	CAAGGTACCGGGGTCACAATCCAAATATGTTGTG	KpnI
jcdA-5'r	AAAAAGCTTCATATTAACATGTACCTCACGATCACCA	HindIII
jcdA-3'f	TCAGGATCCTCGGTTGGATTCACTATGTTCATAATATTG	BamHI
jcdA-3'r	ATTGCGGCCGCGAAATTGCTGGAAATTATACAGGTA	NotI
jcdA_negF1	GCAATTGAAAAATGGACACCAG	
jcdA_negR1	CTTTACAACCAATAAATGAACCC	
jcdA_negF2	CATTGTTTTAATTGCACCATCTG	
jcdA_negR2	AGGGTAATGTTATGATGATGATGC	
jcdA_posR	TGTCAATTCCAATGGTACCAATAATGCC	
mybN-5'f	TTTAGGTACCCAATCAACCTTTTTATTTTAAATC	KpnI
mybN-5'r	GTAAAAGCTTTGAAAATGGTGTAGATGTTGA	HindIII
mybN-3'f	TAGGGGATCCTTGGATGGATTTATCAAAAGC	BamHI
mybN-3'r	AAATGCGGCCGCTCTGTCTATATAATGTTGATG	NotI
mybN_negF1	AACATCTTCCTCATCGATTTA	
mybN_negR1	AGAGATTGATTTTGACCATTACCA	
mybN_negF2	CAACAAGTAGAGGATTAAGAAATC	
mybN_negR2	TAGGTATTTTAGTTTGTCTTGCTC	
mybN_posR	CTCTTTTGAACTCCAATTCGTTGGT	
275333_A	TTTGGGTACCCATATGAAGCATTGCTCGGTG	KpnI
275333_B	TTCCAAGCTTTCATCAACTGGTTGAATATTGGGACTA	HindIII
275333_C	CCATGGATCCCAGAGTTGGTTTCAGCAGTTATTCATC	BamHI
275333_D	TCAAGCGGCCGCTCGTCAATTAGAAAATGAAG	NotI
275333_negF1	GGAAACCAACAACAAAATAATGG	
275333_negR1	ACTTTGGTGATGATTTTGAGG	
275333_negF2	ACCATCACCTTCAAATGTT	
275333_negR2	TCTTATCCTTGAGGATTCACTT	

Biology Open • Supplementary information

275333_posR	GGTGGTAATGGATTGGATTCTTCGG	
gtaJKO5'F	GGTACCATCAATGTCAACCGCAGCC	KpnI
gtaJKO5'R	AAGCTTGTGGAGAGGGAATTGTTGGAC	HindIII
gtaJKO3'F	GGATCCATACCCTTTGTAATGCATGTGG	BamHI
gtaJKO3'R	GCGGCCGCATGGTATTGATTACCCAGTTGAG	NotI
gtaJ_NegF	GTCCAACAATTCCCTCTCCAC	
gtaJ_NegR	GATTGCTGTATTGCTGTTAACG	
gtaJ_PosR	GATGTGTGGTTGTTGTGGATC	
mybAA-5'f	CACGGTACCACATTCAATATCACTCACTCC	KpnI
mybAA-5'r	TGTAAGCTTGAATTTGATGTTACTGATGTTTGATAGT	HindIII
mybAA-3'f	AAAGGATCCGACTTCTCATCGTTGCCACCAATC	BamHI
mybAA-3'r	ATTGCGGCCGCATATGTGAGGTTTCTAATTTTAGA	NotI
mybAA_negF1	AATGGAATTAACAACTGAAGCC	
mybAA_negR1	CAGTTTAATATTGGTGATGGAACTG	
mybAA_negF2	GACACATCATCCTCCTCATCC	
mybAA_negR2	TGAATCAGTTGGAGTTGACAAAG	
mybAA_posR	GTTTAAGAGAATATTGGGACACAAGGTTC	
mybM-5'f	TATGGTACCCTCCACAAAATCTCACATATAC	KpnI
mybM-5'r	GTTAAGCTTCTTCTTCTTCTGTCCATTTAT	HindIII
mybM-3'f	TCAGGATCCAGTGATAATAGTAGTGATGACGAC	BamHI
mybM-3'r	AAAGCGGCCGCCAATCACCCAAATACTTTATC	NotI
mybM_negF1	GGATGATGAATTTTCAGATAATGAA	
mybM_negR1	TTCTCTTGATTGAAGTGATTTCA	
mybM_negF2	GTATTAGACAACCATCTCCATCA	
mybM_negR2	TTCAATTGAAGATTGATGTGGTGAAG	
mybM_posR	CATTTATGGATCAATTTGAATTAACATTTAATCAATATC	
BsrF	GCCGCATGGTTAATTCCTGCAG	
LoxP3'F	GCTCGGATCTGATATCATAACTTC	

Biology Open • Supplementary information

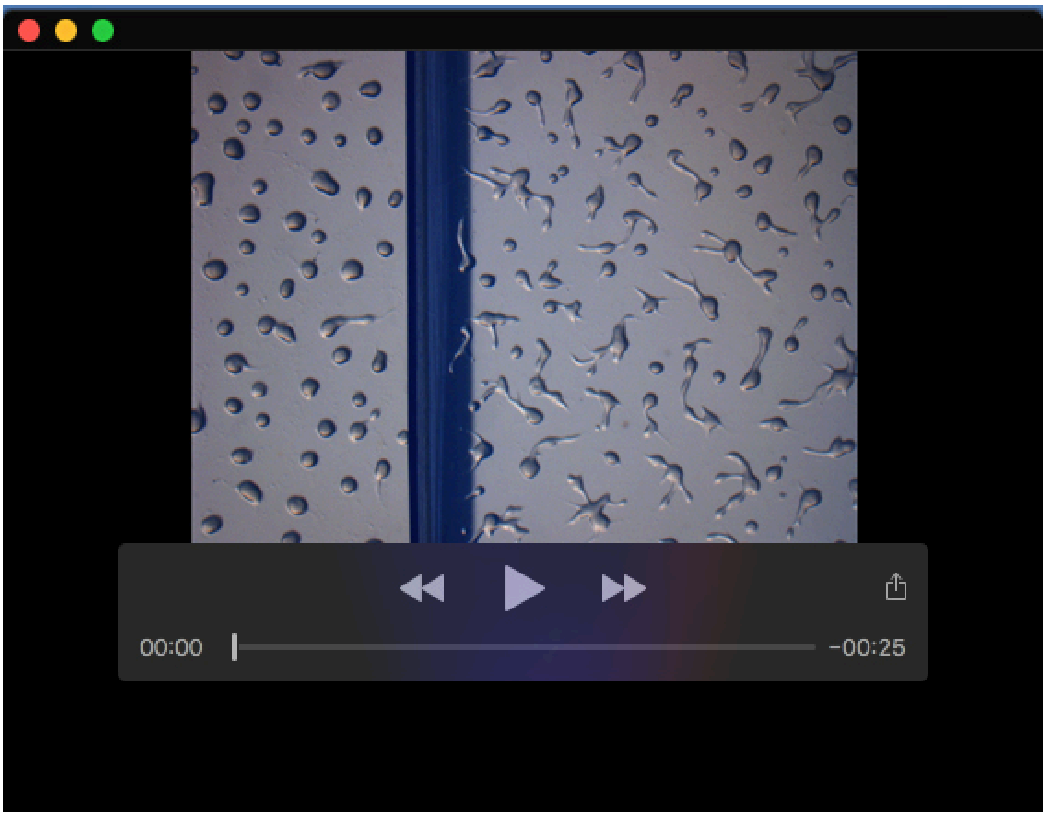

Movie 1. Development of *mybAA*⁻ and wild-type cells

mybAA- and wild-type cells were grown in axenic medium, harvested and plated on NN agar plated at 1.5 x 10^6 cells/cm^2 on NN agar, separated by a narrow strip of overhead sheet inserted in the agar, with wild-type cells on the left and *mybAA*⁻ cells on the right. Images were taken every 5 min for 18 h until fruiting structures had formed, using a Leica stereomicroscope. The movie was compiled at 30 frames per second.

Biology Open • Supplementary information
